# Hip Osteoarthritis and Osteoporosis: Clinical and Histomorphometric Considerations

**DOI:** 10.1155/2014/372021

**Published:** 2014-04-14

**Authors:** Umberto Tarantino, Monica Celi, Cecilia Rao, Maurizio Feola, Irene Cerocchi, Elena Gasbarra, Amedeo Ferlosio, Augusto Orlandi

**Affiliations:** ^1^Department of Orthopaedics and Traumatology, University of Rome “Tor Vergata”, Viale Oxford 81, 00133 Rome, Italy; ^2^Department of Anatomic Pathology, University of Rome “Tor Vergata”, Viale Oxford 81, 00133 Rome, Italy

## Abstract

Although an inverse relationship between osteoarthritis (OA) and osteoporosis (OP) has been shown by some studies, other reports supported their coexistence. To clarify this relationship, we analyzed the interplay between clinical and histomorphometric features. Bone mineral density (BMD) and histomorphometric structure were assessed in 80 patients of four different age-matched groups undergoing hip arthroplasty for severe OA or OP-related femoral fracture. Harris Hip Score was also performed. Surgical double osteotomy of the femoral head was performed and microscopic bone slice samples analysis was performed by using a BioQuant Osteo software. Bone volume fraction (BV/TV) was lower (*P* < 0.01) in subjects with femoral neck fracture (20.77 ± 4.34%) than in subjects with nonosteopenic OA (36.49 ± 7.73%) or osteopenic OA (32.93 ± 6.83%), whereas no difference was detected between subjects with femoral neck fractures and those with combined OA and OP (20.71 ± 5.23%). Worse Harris Hip Score was found in those patients with the lowest BMD and BV/TV values. Our data support recent evidences indicating the possibility of impaired bone volume fraction in OA patients, with a high risk of developing OP, likely for their decreased mobility. Further studies are needed in order to investigate biomolecular pathway and/or growth factors involved in bone volume impairment in OA patients.

## 1. Introduction 

The improvement of living conditions and advances in medicine in the last 50 years increased life expectancy, allowing ageing-related diseases to become a common cause of death and disability. Osteoarthritis (OA) and osteoporosis (OP) are extremely frequent among elderly people and their impact on life quality makes them of high sociohealth relevance [1–3]. Several observations reported an inverse association between OA and OP and large longitudinal studies suggested a protective effect of one disease on the other one [4–7]. This belief was partly supported from the evidence of opposite mechanisms driving the development of bone changes associated with OA and OP. Reduction of the bone mass and quality are key features of OP and determine a high risk of fractures [8, 9]. Instead, OA is characterized by increased bone density [10–14] and cartilage remodelling opposite to those of OP [10, 14–16]. However, other studies failed to show an inverse relationship between OA and OP and reported impaired bone quality and increased risk of fracture in patients with OA [17–20]. Histomorphometry is a recently developed method aimed at evaluating microscopic structure of bone that reflects changes and turnover activities of absorption and formation [21]. Histomorphometric assessment allows for a comprehensive semiquantitative analysis of microscopic organization and structure of bone by using specific grids and software. This allows a computer-assisted analysis of images and obtaining detailed information on volumes and surfaces occupied by different bone component, with particular reference to the distribution of the bone volume compared to the total area. In order to better clarify the relationship between OA and OP, we compared clinical and microscopic bone features in patients with OA or fracture undergoing hip arthroplasty. Dual energy X-ray absorptiometry (DXA) and histomorphometry were used in different subgroups of patients to evaluate bone mass and microarchitectural bone parameters, respectively. The comparative analysis gave better comprehensive information on the relationship between hip OA and OP and the hypothetical mechanisms underlying this association.

## 2. Materials and Methods

### 2.1. Selection of Patients

From June 2011 to September 2012, 119 patients underwent hip arthroplasty in the Orthopaedic Department of Tor Vergata University; patients' written consensus was obtained. Before surgery, each patient with OA underwent DXA examination of the lumbar spine and femoral neck on the same limb on which the operation was planned to estimate the bone mineral density (BMD) and the possible condition of OP according to WHO criteria [22]. Hip X-rays were taken to establish the grade of OA; spine X-rays were also performed in patients with femoral fracture or back pain to evaluate the presence of a vertebral compression fracture (VCF). Lumbar spine and nonfractured femur BMD were also evaluated few days after the surgery. To evaluate hip function, Harris Hip Score (HHS) was also calculated. It gives a maximum of 100 points; the higher the HHS, the less the dysfunction. Exclusion criteria were as follows: history of primary or secondary malignant bone tumors, smoking habit, alcohol abuse, diabetes, hypercholesterolemia and use of glucocorticoids, and a previous fracture on the same or contralateral femur. Patients did not take antiosteoporotic drugs. Among fractured group, 7 patients received a supplementation of calcium and vitamin D.

Four different groups were made according to BMD results and principal diseases (i.e., femoral neck fracture and OA) by enrolling 20 consecutive age-matched patients responding to the following criteria: (1) hip OA and T-score greater than −1 DS (group OA-norm); (2) hip OA and T-score between −1 and −2.5 DS, a condition indicative of osteopenia (group OA-op); (3) hip OA and T-score less than −2.5 DS, a condition indicative of osteoporosis (group OA-OP); (4) femoral neck fracture and T-score less than −2.5 DS, a condition indicative of OP (group FX-OP). Differences among the data of the four groups were analyzed, and their significance was evaluated by Student's *t*-test. In general, *P* values less than 0.05 were considered statistically significant.

### 2.2. Evaluation of Bone Mineral Density

BMD was evaluated by iDXA (Lunar, GE Healthcare, Diegem, Belgium). Lumbar spine (L1–L4) and femoral (neck and total) scans were performed, and BMD was measured (in grams per square centimeter) and analyzed as just described. The coefficient of variation percentage (CV%) of lumbar spine (L1–L4) and proximal femur was 1.1% and 0.7%. Additional quality controls were done every morning for the DXA equipment according to the manufacturer's guidelines, to verify the stability of the system, and did not show any shift during the entire study period. In all groups, measurements were performed while participants lay supine on an examination table with their limbs abducted away from the trunk.

### 2.3. Preparation and Analysis of Specimens

During surgery, a 5 mm thick sagittal slice was obtained from the femoral head. Samples were fixed in 10% buffered formalin and subsequently decalcified in Decalcifier II (Surgipath, Leica Microsystems Srl., Milan Italy) [23]. Successively, after accurate sampling of all slices, samples were dehydrated in increasing concentrations of ethanol and embedded in paraffin. Serial 5 *μ*m thick sections were cut, placed on positively charged slides, and stained with haematoxylin and eosin for microscopic examination [24].

### 2.4. Histomorphometric Analysis

For each femoral head, we evaluated at least 15 microscopic images randomly selected from at least three bone slides, for a total of 15 acquisitions per patient. Images were selected at 40x magnification by using a Nikon Eclipse E600 light microscope connected to a Nikon digital camera and saved at a resolution of 1280 × 1024 pixels. Successively, the images were analyzed by using a BioQuant Osteo software (version 7.20.10; BIOQUANT Image Analysis Corporation, Nashville, USA), specific for histomorphometric bone analysis, according to the manufacture's suggestions. Among the results contained in the BioQuant Osteo software report, we considered the bone volume fraction as percentage of bone volume/total volume ratio (BV/TV), corresponding to the percentage of the bone in the examined surface/field.

## 3. Results and Discussion

**Figure 1:**
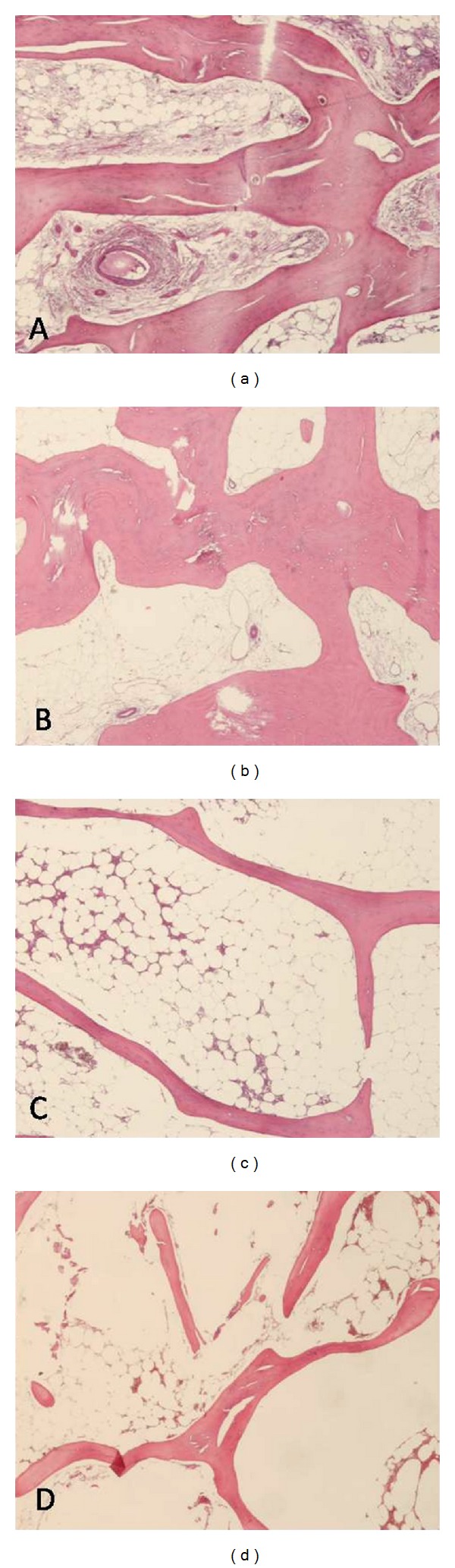
Example of microscopic images acquired from each group of patients. (a) Patients with osteoarthritis (OA) and normal bone mineral density (BMD), (b) OA patients with osteopenic BMD, (c) OA patients with osteoporotic BMD, and (d) fractured patients with osteoporotic BMD. The latter shows a greater separation among bone trabeculae that appear thinner in patients with osteoporotic BMD than in those with normal or osteopenic BMD. Haematoxylin and eosin staining, original magnification ×40.

Examples of acquired fields of microscopic bone structure of four age-matched different groups are reported in Figure 1 and clinical features are summarized in Table 1. Regarding the hip functional assessment, OA patients with normal or osteopenic BMD values displayed a higher HHS score (mean value 41.2 ± 8.6 and 33.4 ± 7.2, resp.) compared with osteoporotic OA patients (mean score 25.5 ± 7.6). Spine X-ray examination documented a VCF in 8 patients (40%) with femoral neck fracture, in line with the literature [25]; 4 patients with OA complained of back pain, but only one patient with osteoporotic BMD displayed a VCF.

Histomorphometric analysis of femoral head samples (Table 1) highlighted significant differences in BV/TV between fractured patients and OA patients with normal BMD (*P* < 0.0001) and between fractured patients and OA patients with osteopenic BMD (*P* < 0.0001), while there was no significant difference between fractured and osteoporotic OA patients (*P* = 0.975), neither between OA patients with normal or osteopenic BMD (*P* = 0.192). The identification of a subset of OA patients with osteoporotic or osteopenic BMD suggested that OA and OP can coexist in some cases, with no evident protective role of one disease on the other one. Our results also highlighted a good correlation between clinical score and histomorphometric features. The reduced BV/TV in OA patients suggests a potential risk for the development of OP. Decreased mobility of patients due to severe OA could be able to impair bone quality and probably increase the risk of fracture. Nevertheless, biomolecular pathways involved in the reduction of BV/TV in OA patients are largely unknown. Many factors or biomarkers have been evocated to mediate bone tissue remodelling. Upregulation of VEGF and its receptors has been shown to be expressed in OA cartilage [26]. Deep chondrocytes normally express antiangiogenic protease inhibitors and their failure can facilitate angiogenic-mediated cartilage remodelling and bone deposition [27]. Insulin growth factor-1 (IGF-1) is the most abundant growth factor in the bone matrix and maintains bone mass in adults. Recently, it has been reported that IGF-1 is markedly decreased in osteoporotic bone in old subjects [28], suggesting a main role of mesenchymal stem cells in the Akt/mTOR pathway mediated bone homeostasis, as also documented for other tissues [29]. During osteogenesis, mesenchymal stem cells also overexpress sortilin [30], and its age-related reduction may cause bone volume loss associated with OP and also pathological vascular remodelling [30, 31]. A better knowledge of mechanisms regulating growth factor expression may suggest new therapeutic opportunities [32, 33] in selected subgroups of OA patients.

The main limit of the present study was the relatively small cohort of patients, which deserves the investigation of additional cases to reinforce the present conclusions. Another limiting aspect was the mainly observational nature of the study, which should be integrated from the analysis of involved tissue growth factor expression to explain the different behaviour of OA patients also developing OP.

## 4. Conclusions

It remains an unsolved question whether OA and OP are related or not. In this study, we addressed this problem combining clinical and structural features from OA or fractured patients. Our preliminary data support the hypothesis that hip OA and OP can coexist, for the presence of a specific subgroup of OA patients with reduced BV/TV and high risk of developing OP.

## Figures and Tables

**Table 1:** Comparison of clinical and histomorphometric parameters in the 4 different age-matched groups of patients with hip osteoarthritis and osteoporosis.

	Osteoarthritis	Osteoarthritis + osteopenia	Osteoarthritis + osteoporosis	Femoral neck fracture
Number of patients (male/female)	11/9	11/9	9/11	8/12
Age (years)	71.7 ± 5.2	71.6 ± 7.2	73.0 ± 4.5	71.9 ± 9.0
Harris Hip Score	41.2 ± 8.6	33.4 ± 7.2	25.5 ± 7.6	73.9 ± 13.2*
Bone mineral density (*T*-score)	0.89 ± 0.85	−1.32 ± 0.84	−2.57 ± 0.51	−2.67 ± 0.51
Bone volume fraction (BV/TV) %	36.49 ± 7.73	32.93 ± 6.83	20.71 ± 5.23	20.77 ± 4.34

Values are ±SD; *calculated on the contralateral femur. *P* values are reported in the text.
